# Analysis of Overactive Bladder Symptom Score Improvement in Lower Urinary Tract Symptom Patients During Behavioral Therapy While Using the Smartphone Application "USAPO"

**DOI:** 10.7759/cureus.82406

**Published:** 2025-04-16

**Authors:** Kosuke Mikami, Kanya Kaga, Tomohiko Ichikawa

**Affiliations:** 1 Department of Urology, Chiba University Hospital, Chiba, JPN

**Keywords:** behavior therapy, health records, lower urinary tract symptoms, mobile applications, overactive bladder

## Abstract

Introduction: According to Japanese lower urinary tract symptom (LUTS) guidelines, behavioral therapy is recommended as a first-line treatment for LUTS. However, the guidance provided during outpatient visits alone may not be sufficiently effective, as patients are unlikely to change their behavior. This study aimed to evaluate the changes in urinary storage symptoms after sustained intervention using a mobile application.

Methods: Changes in Overactive Bladder Symptom Score (OABSS) resulting from the sustained intervention were assessed among users of the mobile application "USAPO."

Results: A total of 139 patients were included in this study. The OABSS before and after using USAPO was 2.03±2.42 and 2.25±2.826 points, respectively, with no significant difference (p=0.2194). The group with at least a one-point improvement in OABSS showed a statistically significant trend in age (p=0.055) and a statistically significant difference in the severity of urinary storage symptoms compared to the non-responding group (p<0.0001).

Conclusions: Although no overall change in OABSS was observed with the introduction of USAPO, a subset of patients may benefit from its use.

## Introduction

When symptoms are present in the lower urinary tract, which consists of the bladder and urethra, it is called lower urinary tract dysfunction (LUTD) and causes various lower urinary tract symptoms (LUTS) related to urine and urination.

According to an online survey conducted in Japan in 2023, the prevalence of LUTS was 77.9% (males: 79.4%, females: 76.5%) among participants aged ≥20 years and 82.5% (males: 85.0%, females: 80.1%) among those aged ≥40 years [1]. This corresponds to over 80 million individuals with LUTS as their primary complaint. The number of patients with LUTS tends to increase with age and is expected to increase further as the population ages [2].

LUTS include urinary storage, voiding, and post-urinary symptoms. Although urinary storage symptoms rarely cause life-threatening upper urinary tract disorders or other physical disabilities compared to voiding symptoms, they significantly impair quality of life [3].

Although verbal guidance and pamphlets as the primary treatment are commonly used in outpatient settings [4], they may be insufficient for achieving therapeutic effects. Daily outpatient care alone often fails to demonstrate the effectiveness of behavioral therapy.

To promote lifestyle modifications and enhance symptom management, Welby, Inc. (Tokyo, Japan) developed the USAPO smartphone application, a personal health record (PHR) system. This application was included in the second edition of the Nocturia guidelines. It enables users to record their urinary storage symptoms, urination frequency, and urine volume using the Overactive Bladder Symptom Score (OABSS) and a urinary diary. Users can also take pictures of their diet; estimate their salt, alcohol, and caffeine intake; and receive tailored lifestyle advice. This approach aims to enhance the overall effectiveness of behavioral therapy by providing personalized guidance. We hypothesize that the use of "USAPO" may improve the effectiveness of behavioral therapy, which is insufficient when limited to brief outpatient instruction.

In this study, we evaluated estimated salt, alcohol, and caffeine intake among "USAPO" users and retrospectively examined changes in OABSS before and after the advice was provided by the application.

## Materials and methods

All scores and data recorded by the "USAPO" were used for the analysis. The study protocol was approved by the Ethics Review Board of Chiba University (reception number: M10667) and conducted in accordance with the principles of the Declaration of Helsinki. Consent to participate was obtained in an opt-out format, as this was a retrospective study.

Study design and patients

Data were collected from 2019 to 2021 and extracted from the "USAPO" smartphone application (Welby, Inc., Tokyo, Japan). Participants included "USAPO" users who reported urinary urgency as their primary complaint, recorded dietary data for at least three days (including three meals per day), and documented OABSS at least twice.

The OABSS assesses urinary storage symptoms, including overactive bladder (OAB), and uses a four-item questionnaire with a total score of 0-15 points: Q1, 0-2 points; Q2, 0-3 points; Q3, 0-5 points; and Q4, 0-5 points [5]. Disease severity was classified as mild (<5 points), moderate (6-11 points), or severe (≥12 points) [6,7].

The primary end point was the change in the total OABSS before and after behavioral therapy in "USAPO" users. Secondary analyses included comparisons of salt, alcohol, and caffeine intake, as well as patient demographics such as age, sex, body mass index (BMI), height, and OAB severity between the responder and non-responder groups. Responders were defined as those with at least a one-point improvement in the OABSS after the intervention.

USAPO application software

**Figure 1:**
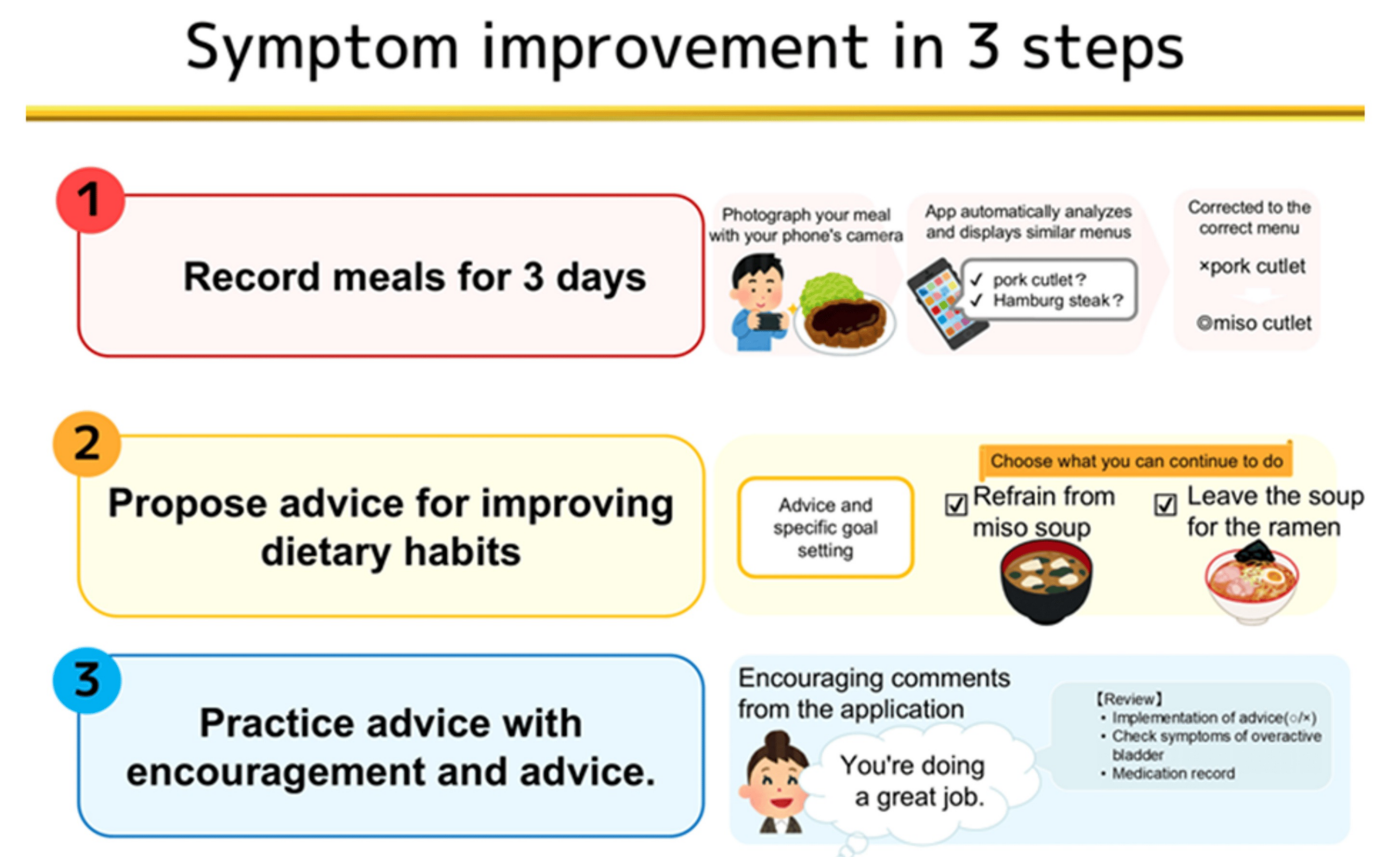
USAPO offers three steps to help patients achieve dietary improvements 1. The menu can be easily input by taking a picture of the meal with a smartphone camera and using the analysis function. The amount of salt, caffeine, and alcohol expected from the menu is automatically calculated. Meals can be recorded in five categories: breakfast, lunch, dinner, bedtime, and snacks. 2. Based on the information recorded in Step 1, we will provide you with dietary improvement advice. The advice is divided into three categories: salt, caffeine, and alcohol. Multiple suggestions are offered according to individual dietary habits, such as "Eat fewer pickles," "Drink one less cup of coffee," and "Take a day off from drinking once a week." 3. You can keep track of whether or not you have achieved each goal. Image credits: Kosuke Mikami

The "USAPO" application was developed by Welby, Inc., with content supervised by urologists from various facilities. Available for both iOS and Android users, the application allows users to record their OABSS, urinary diaries, and meal photos. Participants downloaded the application from the App Store or Google Play; initially registered their date of birth, sex, height, and weight; and recorded their OABSS, urinary diaries, and photos of their meals. Salt, alcohol, and caffeine intake were calculated from the recorded food photos. This information was anonymized and stored in Welby, Inc.'s cloud system. The advice is divided into three categories (salt, caffeine, and alcohol), and multiple pieces of advice are suggested according to individual dietary habits, such as "Eat fewer pickles," "Drink one less cup of coffee," or "Have a day off once a week." When users record their progress, encouraging comments are displayed to motivate them to improve their lifestyles. For convenience, the text of the application was displayed in English (Figure 1).

Statistical analyses

Patient characteristics are expressed as the mean±standard deviation of each continuous variable. All statistical analyses were performed using JMP® 17 (JMP Statistical Discovery, LLC., Cary, NC). The chi-square and t-tests were used for continuous variables, and the Wilcoxon test was used for discontinuous variables. Intergroup differences in categorical variables were assessed using the chi-square test. Statistical significance was set at p<0.05.

## Results

**Figure 2:**
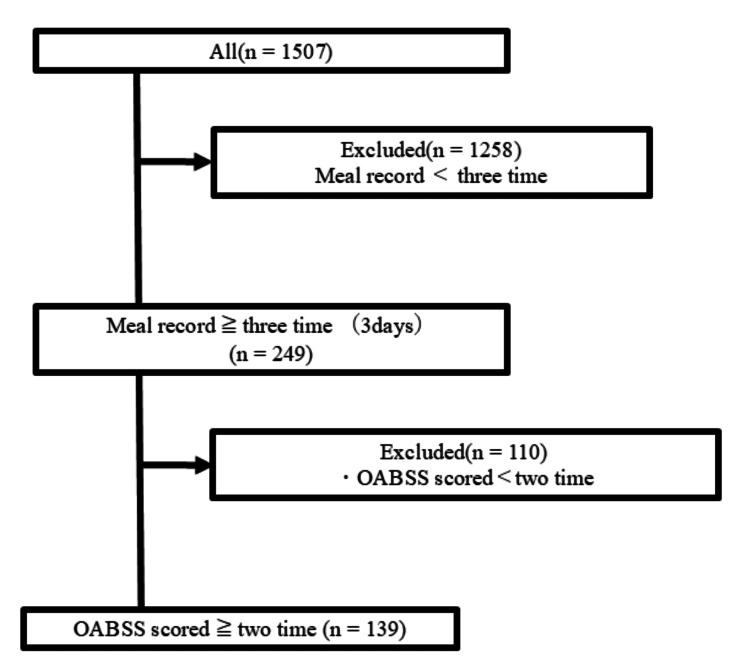
Patient selection OABSS: Overactive Bladder Symptom Score

Among 1,507 patients with a chief complaint of urinary urgency who used "USAPO," 249 patients recorded three meals per day for at least three days. Of these, 139 patients recorded OABSS at least twice and were thus included in the study (Figure 2).

Patient characteristics are shown in Table 1.

**Table 1:** Patient demographics BMI: body mass index

Demographics	Number (%)/median (range) (N=139)
Age (years)	47 (24-75)
Female	37 (26.6%)
BMI (kg/m^2^)	22.2(16.7-43.4)
First	
Light	80 (57.6%)
Moderate	56 (40.3%)
Severe	3 (2.2%)
Final	
Light	77 (55.4%)
Moderate	49 (35.3%)
Severe	13 (9.4%)

Of these, 37 (26.6%) patients were female, with a median age of 47 years (range: 24-75 years) and a BMI of 22.2 kg/m^2^ (range: 16.7-43.4 kg/m^2^). The first recorded OABSS was mild in 80 (57.6%) patients, moderate in 56 (40.3%) patients, and severe in three (2.2%) patients. The details of the OABSS are shown in Table 2.

**Table 2:** OABSS changes before and after USAPO use OABSS: Overactive Bladder Symptom Score, SD: standard deviation

OABSS	First score (mean±SD)	Final score (mean±SD)
Q1	0.44±0.51	0.45±0.55
Q2	0.68±0.91	0.67±0.91
Q3	0.68±1.11	0.72±1.10
Q4	0.23±0.63	0.41±0.87
Total	2.03±2.42	2.25±2.83

The mean±standard deviation of the initial record was 0.44±0.51 points in Q1, 0.68±0.91 points in Q2, 0.68±1.11 points in Q3, and 0.23±0.63 points in Q4, with a total of 2.03±2.42 points. The final record results were 0.45±0.55 points in Q1, 0.67±0.91 points in Q2, 0.72±1.10 points in Q3, and 0.41±0.87 points in Q4, with a total of 2.25±2.83 points. A paired t-test of the corresponding data before and after use revealed no significant differences (p=0.32).

Only eight cases recorded the amount of change in urination records and OABSS (Table 3).

**Table 3:** Recorder of urination diary BMI: body mass index, OAB: overactive bladder

	Gender	Age (years)	BMI (kg/m^2^)	Record days	Average urine volume (mL/day)	Days of record criteria: ≥1	OAB severity
A	Male	54	20.1	6	523.3	Non-responder	Light
B	Female	50	19.6	1	150.0	Responder	Moderate
C	Male	25	22.4	2	950.0	Responder	Light
D	Male	47	30.4	1	150.0	Responder	Light
E	Male	41	25.6	2	1075.0	Non-responder	Light
F	Male	70	21.2	8	1987.5	Responder	Moderate
G	Male	41	19.4	1	200.0	Non-responder	Moderate
H	Female	68	17.4	12	1654.7	Responder	Light

Five patients had records of drinking water intake and OABSS (Table 4).

**Table 4:** Recorder of drinking water BMI: body mass index, OAB: overactive bladder

	Gender	Age (years)	BMI (kg/m^2^)	Record days	Average fluid intake (mL/day)	Days of record criteria: ≥1	OAB severity
I	Male	54	20.1	3	566.7	Non-responder	Light
J	Female	50	19.6	1	500.0	Responder	Moderate
K	Male	25	22.4	2	3050.0	Responder	Light
L	Male	70	21.2	7	1571.4	Responder	Moderate
M	Female	68	17.4	9	1407.8	Responder	Light

The results for the responders and non-responders are shown in Table 5.

**Table 5:** Patient demographics in the responding and non-responding groups after using USAPO BMI: body mass index, OAB: overactive bladder, OABSS: Overactive Bladder Symptom Score

Demographics	OABSS non-responders (n=90)	OABSS responders (n=49)	p
Age (years)	46 (26-75)	53 (24-75)	0.0553
Female (number (%))	21 (18.9%)	16 (32.7%)	0.3152
BMI (kg/m^2^)	22.5 (17.0-29.4)	22.4 (20.8-24.4)	0.8329
OAB severity			
First			
Light (number (%))	69 (76.7%)	11 (22.4%)	<0.0001*
Moderate (number (%))	21 (23.3%)	35 (71.4%)	
Severe (number (%))	0 (0%)	3 (6.1%)	
Final			
Light (number (%))	49 (54.4%)	28 (57.1%)	0.2786
Moderate (number (%))	39 (43.3%)	19 (38.8%)	
Severe (number (%))	11 (12.2%)	2 (4.1%)	

The number of responders and non-responders was 49 and 90, respectively. The ages (mean±standard deviation) of responders (50.7±16.0 years) tended to be older than those of non-responders (46.1±11.9), but this was not significant (p=0.0553). There was no significant difference in sex and BMI between the two groups (p=0.3152 and p=0.8349, respectively). Regarding the severity of urinary symptoms, 11 (22.4%) responders and 69 (76.7%) non-responders had mild symptoms, 35 (71.4%) responders and 21 (23.3%) non-responders had moderate symptoms, and three (6.1%) responders and 0 (0%) non-responders had severe symptoms, with significant differences observed between the two groups (p<0.0001).

Table 6 shows the results of dietary intake.

**Table 6:** Comparison of diet recording between the improving and non-improving groups OABSS: Overactive Bladder Symptom Score, SD: standard deviation

All (N=139)				OABSS non-responders (n=90)		OABSS responders (n=49)		p
Variables		Number	Mean±SD	Number	Mean±SD	Number	Mean±SD	
Calorie (Kcal)	Breakfast	138	351±162.09	89	336.617±162.66	49	377.124±159.373	0.1609
	Lunch	139	603.635±209.05	90	612.7688±223.908	49	586.859±179.559	0.4871
	Dinner	137	672.037±220.912	88	675.848±228.965	49	665.193±207.794	0.7878
	Snacking	99	172.669±172.688	58	176.324±180.154	41	167.5±163.602	0.8037
	Bedtime snack	59	173.69±142.567	36	159.413±94.573	23	196.039±196.215	0.3402
Total	All	139	1811.184±482.38	90	1783.8733±481.153	49	1861.349±485.553	0.3675
	Breakfast, lunch, and dinner	139	1614.479±430.854	90	1606.476±446.577	49	1629.177±404.453	0.7678
	Dinner and bedtime snack	108	229.466±189.975	65	206.246±169.463	49	264.567±214.684	0.1188
Salt (g)	Breakfast	138	0.736±0.76	89	0.659±0.744	49	0.892±0.773	0.0845
	Lunch	139	2.927±1.625	90	2.874±1.546	49	3.025±1.772	0.6021
	Dinner	137	2.238±1.322	88	2.342±1.398	49	2.141±1.113	0.3886
	Snacking	99	0.228±0.83	58	0.244±0.938	41	0.207±0.658	0.8287
	Bedtime snack	59	0.189±0.462	36	0.208±0.468	23	0.16±0.461	0.7043
Total	All	139	6.082±2.132	90	6.058±2.223	49	6.309±2.467	0.5417
	Breakfast, lunch, and dinner	139	5.97±2.228	90	5.817±2.241	49	6.06±2.328	0.5481
	Dinner and bedtime snack	137	2.54±1.59	88	2.405±1.472	49	2.217±1.142	0.4404
Caffeine (g)	Breakfast	139	34.975±44.7	90	34.715±43.32	49	35.453±47.586	0.9264
	Lunch	139	9.117±15.834	90	9.666±16.483	49	8.11±14.679	0.5819
	Dinner	139	1.737±5.742	90	2.34±6.676	49	0.63±3.19	0.0937
	Snacking	135	40.72±50.62	86	38.441±50.437	49	44.719±51.212	0.4904
	Bedtime snack	132	3.35±16.326	84	1.815±12.159	48	6.364±21.685	0.1538
Total	All	139	88.561±77.31	90	85.15±73.241	49	94.828±84.708	0.4827
	Breakfast, lunch, and dinner	139	45.831±50.648	90	46.722±51.058	49	44.195±50.371	0.7798
	Dinner and bedtime snack	139	4.919±16.845	90	4.034±13.697	49	6.544±21.536	0.4033
Alcohol (g)	Breakfast	139	0	90	0	49	0	0
	Lunch	139	0.1139±0.973	90	0.1759±	49	0	0.3107
	Dinner	139	5.434±9.049	90	5.668±	49	5.003±8.704	0.6804
	Snacking	135	0.432±2.174	86	0.498±	49	0.316±1.57	0.6423
	Bedtime snack	131	2.594±6.435	84	2.982±	47	1.898±4.819	0.3572
Total	All	139	13.96±20.913	90	14.949±	49	12.144±19.635	0.452
	Breakfast, lunch, and dinner	139	5.548±9.327	90	5.844±	49	5.003±8.704	0.6132
	Dinner and bedtime snack	139	7.878±11.49	90	8.452±	49	6.824±10.958	0.427

No significant differences in caloric, salt, caffeine, or alcohol intake were found between the two groups.

## Discussion

The "USAPO" PHR application, provided by Welby Inc., provides feedback to patients on their salt, caffeine, and alcohol intake by having them enter their dietary records and OABSS data. The goal of this application is to improve urinary symptoms, such as overactive bladder and nocturnal polyuria, through behavioral changes in eating and drinking habits.

PHR applications are believed to promote patient-centered care by serving as a tool for behavioral change. Several studies have demonstrated the efficacy of mobile PHR interventions in fields such as oncology, where intervention groups have shown prolonged survival rates [8]. Furthermore, reports indicate that in patients with arrhythmia and heart failure, PHR intervention groups have improved life outcomes compared to the usual care group, owing to earlier detection of arrhythmias than regular outpatient visits [9].

Two applications have been approved in Japan as of 2024: the Hypertension Treatment Assistance Program (CureApp HT®) and the Smoking Cessation System (CureApp SC®). The CureApp SC improved the rate of continuous smoking cessation in a Japanese phase III multicenter clinical trial [10]. Therefore, we hypothesized that the use of a PHR application could enhance urinary symptom improvement by improving the OABSS and promoting behavioral changes through dietary records and simple lifestyle guidance. However, our study did not observe any improvements in salt, alcohol, or caffeine intake and no associated improvements in the OABSS.

Some reports have suggested that simple restrictions on salt, alcohol, and caffeine lead to improvements in nocturia and frequent urination [1,11]. The low retention rate, inadequate recording, and unenforceable feedback function of the "USAPO" may be among the reasons for the lack of improvement observed in our study. These factors may not have led to spontaneous behavioral changes among patients.

In addition, as the "USAPO" used in this study is a free application currently available to the public, it is likely that many patients did not receive regular interventions by medical personnel. Therefore, it is possible that this did not lead to active behavioral interventions. The low rate of urinary diaries was likely due to the requirement of entering data hourly. In the future, we plan to improve this application to facilitate better engagement.

Notably, this study discusses which patient populations are best suited for promoting lifestyle improvements through this application. A recent report indicated that most male patients visiting healthcare facilities to receive treatment for LUTS were significantly older than 70 years. The current study included 73% of men with a median age of 47 years. Patients in our study were younger than those in the original group of patients with bladder overactivity. Within this patient background, the responder group predominantly included a higher proportion of patients with severe OABSS. The responder group was also slightly older than the non-responder group, although the difference was not statistically significant. This may indicate that symptoms worsen with age in patients with urinary storage symptoms. As the symptom rate tends to increase significantly with age in almost all LUTS [12], we believe that in the future, USAPO could be useful for older users, as well as for those with severe urinary storage symptoms, among those in the patient population who have not sought medical care.

However, internet usage tends to decline with age, and there may be hurdles in understanding and using communication technology. According to the Ministry of Internal Affairs and Communications' Survey on Communications Usage Trends, over the five-year period from 2014 to 2018, internet usage remained at approximately 75%, 50%, and 20% among those aged 60, 70, and 80 years, respectively. Although internet usage rates among those in their 50s-70s are lower than those under 50, the trend is increasing year by year, particularly among those in their 50s. Additionally, even among those aged 60 and over, the current smartphone usage rate has exceeded 80%, and considering that the age group using mobile applications will become even older, the hurdle for application use in the future is expected to be lowered.

Applications for diagnosing overactive bladder and nocturnal polyuria using actual voiding records and the OABSS [13], electronic urine flow meters, and electronic voiding diaries linked to mobile applications have been developed and reported to improve reliability [14].

However, to date, no studies have demonstrated an improvement in behavioral changes in response to urinary symptoms through the use of dietary records. Clarifying the layers of the approach in the present study is a significant development for future prospective studies. In the future, we will consider the influence of additional factors, such as underlying diseases, current medication status, cognitive function, and records of urinary and drinking habits. We also expect that patients will not only record the information but also receive feedback from physicians, nurses, and nutritional therapists, which could lead to voluntary behavioral changes and further improvement of urinary symptoms.

This study has some limitations. It was a retrospective, observational study, and the status of patients' medical visits could not be ascertained due to the lack of detailed records on medication use (e.g., "took medication," "partially took medication," or "did not take medication" for overactive bladder and benign prostatic hyperplasia medications). Additionally, a previous study highlighted the accuracy of dietary records and the differences in accuracy depending on the measured parameters [15]. Therefore, the extent to which dietary records are correlated with clinical efficacy remains unclear. OABSS is a discrete variable with a range of 0-15 points and is not continuous data. Since normal distribution assumes a continuous variable, OABSS is difficult to assume a normal distribution and has a skewed distribution, so consider that the SD is larger than the mean.

## Conclusions

In this study, we aimed to promote behavioral therapy in patients with urinary symptoms using the "USAPO" electronic urinary drainage diary, which records dietary details. Although the intervention using "USAPO" did not lead to significant changes in the OABSS, our findings suggest that "USAPO" may be particularly effective for older patients and those with severe urinary symptoms.

In addition, USAPO can quantify caloric, salt, caffeine, or alcohol intake by recording meals, which may lead to the establishment of evidence for dietary therapy for LUTS, which has been unclear until now.
